# Improving brain B_0_ shimming using an easy and accessible multi-coil shim array at ultra-high field

**DOI:** 10.1007/s10334-022-01014-6

**Published:** 2022-05-05

**Authors:** Vincent Oltman Boer, Jan Ole Pedersen, Nick Arango, Irene Kuang, Jason Stockmann, Esben Thade Petersen

**Affiliations:** 1 Danish Research Centre for Magnetic Resonance, Centre for Functional and Diagnostic Imaging and Research, Copenhagen University Hospital Amager and Hvidovre, section 714, Kettegård Allé 30, 2650 Hvidovre, Hvidovre Denmark; 2 Philips Healthcare, Copenhagen, Denmark; 3 Massachusetts Institute of Technology, Cambridge, MA USA; 4 Department of Radiology, Athinoula A. Martinos Center for Biomedical Imaging, Massachusetts General Hospital, Charlestown, MA USA; 5 Harvard Medical School, Boston, MA USA; 6 Department of Health Technology, Centre for Magnetic Resonance, Technical University of Denmark, Kgs. Lyngby, Denmark

**Keywords:** Magnetic resonance imaging, Ultra-high field, B_0_ field, Shimming, Multi-coil array

## Abstract

**Object:**

Improve shimming capabilities of ultra-high field systems, with addition of an accessible low-complexity B_0_ shim array for head MRI at 7 T.

**Materials and methods:**

An eight channel B_0_ shim coil array was designed as a tradeoff between shimming improvement and construction complexity, to provide an easy to use shim array that can be employed with the standard 7 T head coil. The array was interfaced using an open-source eight-channel shim amplifier rack. Improvements in field homogeneity for whole-brain and slice-based shimming were compared to standard second-order shimming, and to more complex higher order dynamic shimming and shim arrays with 32 and 48 channels.

**Results:**

The eight-channel shim array provided 12% improvement in whole brain static shimming and provided 33% improvement when using slice-based shimming. With this, the eight-channel array performed similar to third-order dynamic shimming (without the need for higher order eddy current compensation). More complex shim arrays with 32 and 48 channels performed better, but require a dedicated RF coil.

**Discussion:**

The designed eight-channel shim array provides a low-complexity and low-cost approach for improving B_0_ field shimming on an ultra-high field system. In both static and dynamic shimming, it provides improved B_0_ homogeneity over standard shimming.

## Introduction

Numerous novel approaches for improving MRI and MRS at ultra-high field have been shown since the introduction of 7 T scanners. One challenge is B_0_ field compensation (shimming) which is required to mitigate field inhomogeneities in the brain caused by the inherent variation in magnetic susceptibility. As susceptibility effects scale with field strength, a better B_0_ field compensation is typically needed at ultra-high field to prevent signal loss, image distortions [1, 2] and data quality loss in, e.g., MR spectroscopy [3]. However, since the introduction of ultra-high field over a decade ago, not much improvement has been made in B_0_ shimming [4]. The standard shimming hardware remains whole body second-order spherical harmonic (SH) shimming, similar to what is available in clinical 3 T systems. Several 7 T systems have been extended with third-order shimming, which provides much needed improvements in shimming, especially in large FOV imaging of the body [5]. Even higher order SH shimming with an insert coil providing 4th and some 5th order SH-terms using an insert coil promises even more gain in field homogeneity in the brain [6].

Apart from increasing the complexity of SH-shim systems, significant improvements in achievable field homogeneity can also be gained with slice-based (referred here as dynamic) shimming. Here the shimming is optimized for only the volume that currently is being imaged and updated for, e.g., each slice read-out [7, 8]. The reduced optimization volume (i.e., only a small stack of slices) leads to better field homogeneity, but entails additional implementation challenges, such as dynamic updating of the scanner’s shims, gradients and center frequency (*f*_0_). Recently, dynamic shimming was even shown for use with a very high-order SH shim insert [9]. However, alternating the shim currents, as needed for updating the shim per slice, induces unwanted higher order eddy currents. Correcting for these requires additional hardware, advanced reconstructions and/or substantial calibration efforts to measure the eddy currents of a system [10–12]. Alternatively, software optimization [13] or shim constraints can be used to minimize large steps between slices [14].

Localized shimming by independently controlled multi-element array coils (MC) may be performed as an alternative or additionally to SH-shimming. Here a number of small (loop) coils are placed close to the subject, and each steered with a separate amplifier. Due to the proximity to the head, highly localized field compensation can be achieved, without inducing significant eddy currents during switching as the small coils are far away from the gradient coils. However, designing and manufacturing these shim arrays is a significant engineering effort. First, it requires a high number of independent shim amplifiers and dedicated interfacing to an MRI scanner. Second, RF coil performance may be negatively impacted by the presence of conducting structures between the coil and the imaged volume. Several MC-shim configurations specifically for brain imaging have been suggested in literature. These include a 6-channel, variable positioning setup [15], a 48-channel MC-array [16] and a 16-channel MC-array [17]. To solve interaction between receive RF coils and shim coils integrated RF and shim coils have been proposed with 8 [18] and 31 channel arrays [19, 20], the latter one later extended with face loops [21]. In addition, subdivided loops [22] and more complex geometric shapes have been proposed [23]. However, limited work has been done on designs that are compatible with a standard high field system environment, and a standard RF coil.

Therefore, the goal of this research paper was to design an MC-array capable of dynamic shimming that is compatible with a standard high field system and a standard RF head coil. This led to the design of an eight-channel MC-array, placed on the outside of the RF coil shield. Improved B_0_ field homogeneity was reaches on top of standard second-order SH-shimming, although not as much as with the more complex high-count arrays. Most benefit was shown for dynamic shimming in multi-slice sequences.

## Materials and methods

All human experiments were performed in accordance with local ethical guidelines and written informed consent was obtained from all participants. Data was acquired with a two-channel volume transmit (Tx) coil and a 32‐channel receiver array (Nova Medical Inc., Burlington, Massachusetts, U.S.A.) using a whole body 7 T MRI system (Achieva, Philips Healthcare, Best, The Netherlands), equipped with up to and including all third-order spherical harmonics, and employing individual ± 10 A amplifiers.

### Numerical optimization of shim coil geometry

B_0_ field maps were acquired in eleven subjects. The B_0_ field map sequence was a 3D gradient echo with field of view (FOV) of 240 × 240 × 116 mm^3^, isotropic voxel size of 3.75 mm^3^, echo times TE1/TE2/repetition time (TR) = 2/3/10 ms, and scan time: 21 s. Brain extraction was done by manually delineating each scan. In-house developed software (MATLAB, the Mathworks, Natick, MA, USA) was used to optimize shim settings for different MC-array geometries using a current-limited linear solver (lsqlin). Calculation times for whole brain shimming were around 100 ms, for slice-based shimming 500–700 ms. For simulation of the magnetic field for the individual shim array elements, a discrete Biot–Savart formulation was used.

**Fig. 1:**
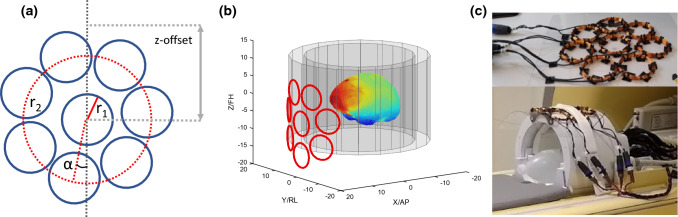
**a** Geometry optimization was performed using a center coil with a ring of coils around it on the outside of the RF coil to minimize coupling. Input parameters to the optimization were the number of coils, the radius of the coil elements (*r*_1_), the radius of the larger ring (*r*_2_), the angle of the outer ring with respect to the main magnetic field direction (*α*) and the z-offset of the array. **b** Final result shown with the inner and outer surface of the RF coil. **c** Realized eight-channel shim array mounted on the RF coil

The designed MC-array geometry was on purpose kept simple to minimize construction complexity, ease handling, and mitigate coupling with the applied RF coil. A geometry was chosen on a cylinder that followed the outside of the RF coil (a cylinder with 37 cm diameter). The MC-array geometry consists of a middle circular shim element with a ring of circular shim elements around it (Fig. 1a). An iterative numerical solver (fmincon) was used to minimize the standard deviation in the head. The solver varied the total number of coils (inner + *n*), radius of the coils (*r*_1_) between 10 and 50 mm, radius of the ring of coils around the center coil (*r*_2_) between 10 and 150 mm, angulation of the outer ring (*α*). The z-offset was limited so it could be fastened on the RF coil housing. The optimization was performed on the B_0_ field map of a single volunteer after removal of second-order SH-fields. To verify that we arrived at a model that is applicable to more than the single subject, the optimization was also performed on ten subjects, to simulate a per-subject design. The achieved field homogeneity was compared to the single-subject optimization.

### Data acquisition and array construction

The MR system was extended with eight inexpensive open-source class-A shim amplifiers [24]. The lead compensator in the amplifier’s feedback loop was adapted to ensure stable operation, with switching speeds below 1.5 ms for 8 channels. The shim amplifiers were supplied with 12 V, facilitating fast switching of ± 2.5 A on each array element. The computer running the shim calculations was also used for compiling code for and flashing of a microcontroller development board (“Teensy 3.5”, PJRC, Sherwood, OR, USA). The microcontroller board was mounted on a custom PCB facilitating trigger-inputs from the scanner and fiber-optic cabling for communication with the shim amplifiers across the faraday cage. Shim settings were pre-loaded to the microcontroller by recompiling inhouse developed code, which took around 1 min. Updating of precompiled slice-based current settings were triggered by a TTL-pulse from the scanner. A ten-meter cable consisting of eight pairwise twisted 0.5 mm^2^ wires was passed from the back of the magnet through the cable-management system of the scanner and connected to the MC-array using four 4-pole speakON connectors. The eight individual circular shim elements were wounded using a 3D printed jig (available from https://resources.drcmr.dk/BrainShimArray) using 50 turns of 0.5 mm^2^ wire. Each element was attached with zip-ties to a flexible acrylic sheet. The sheet was bend into the shape of the outer surface of the RF coil, and the array was embedded in epoxy (West systems, Bay City, USA) to minimize vibration when operated. The array was reproducibly positioned on the RF coil using plastic guiding pins in 3D-printable brackets (available from [25]) to ensure similar placement between experiments, and fastened with Velcro belts.

Calibration shim fields from each individual shim channel were acquired using a large balloon (“Bubble Ball”, unknown manufacturer) containing water and 4 g/L NaCl, which completely filled the internal volume of the receive coil.

Electromagnetic interference between the MC-array and the RF coil was measured from reflection measurements (S11) on the transmit RF coil and by measuring B_1_^+^ maps with and without the shim array in place. To investigate coupling to the receive array the noise correlation matrix was measured with and without the shim array in place.

### Comparison with existing static and dynamic shim techniques

The proposed MC-geometry was evaluated by comparing its simulated performance to other existing shim techniques on B_0_ maps acquired from ten subjects. As a baseline method, we chose to use static second-order shimming, as this is the highest full set of spherical harmonic order available across all 7 T vendors. To minimize off-resonance effects from fat signal contributions brain-segmentation was performed. Both whole-brain shimming and dynamic (slice-based) shimming was evaluated.

For whole-brain shimming the following were compared: (1) second-order SH-shimming and second-order SH-shimming together with (2) the 50-turn eight-channel MC-array, (3) the single-turn 32-channel close fitting MC-array [19] and (4) the 100-turn 48-channel MC-array [10]. The 32- and 48-channel array were available from rflab.martinos.org.

For dynamic shimming on each individual slice, the two neighboring slices were included for through-slice field optimization. The following approaches were compared: (1) *f*_0_ + linear shimming (“first order shimming”), (2) up to and including second-order SH-shimming (“second order shimming”), (3) up to and including third-order SH-shimming (“third order shimming”). For the MC-arrays, dynamic first-order shimming was combined with the (4) 8-channel MC-array, (5) the 32-channel array and (6) the 48-channel array.

Performance between the shimming methods was performed with pairwise paired *t* tests (correcting for multiple comparisons, alpha was set to 0.05/10 = 0.005).

### Dynamic shimming for EPI imaging

An additional subject was scanned using the MC-array for slice-based shimming during EPI. A B_0_ map using the same slice thickness and same number of slices was acquired for calculating a second-order static shim and slice-based shim settings for the eight-channel MC-array with first-order dynamic shimming.

Scanning parameters for the multi-slice EPI sequence were 2 mm in-plane resolution, 240 mm FOV, 2 mm slice thickness, 31 slices, 0.2 mm slice gap, sensitivity encoding (SENSE) = 2, flip angle of 73°, TE/TR = 17/1742, Spectral Presaturation with Inversion Recovery (SPIR) fat suppression and a readout bandwidth of 32 Hz/pixel. As geometric reference, a gradient echo sequence was acquired with similar scanning parameters.

## Results

### Shim array optimization

The numerical optimization resulted in a MC-array consisting of eight coils with a radius (*r*_1_) of 37 mm and peripheral coils position with their center 95 mm from the center of the MC-array (*r*_2_), an angle of 0° to the main magnetic field (*α*) and a z-offset of 8 mm in the caudal direction with respect to the middle of the RF coil housing (Fig. 1b). The subject-specific design performed 1–2 Hz better than the one-subject design, where there was one subject with a larger 6 Hz improvement.

The constructed 50-loop coils of the MC-array had a self-inductance of 303 µH on average (range 295–312 µH) and an electric resistance of 0.2 Ω. The 1m cables added electric resistance of around 1 Ω per channel.

**Fig. 2:**
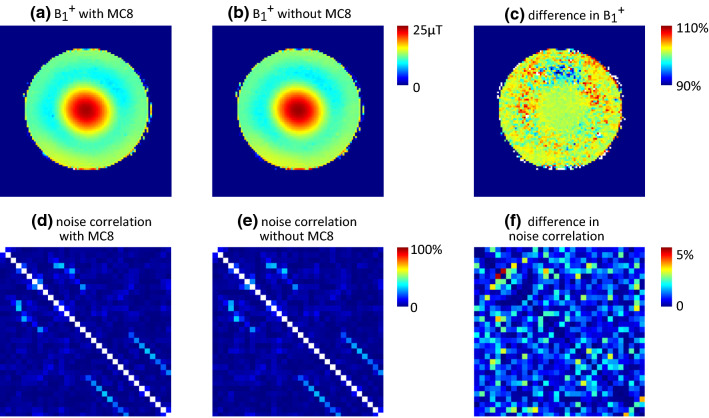
B_1_^+^ mapping **a**–**c** and receiver array noise correlation matrix **d**–**f** with either the eight-channel shim coil (MC8) in place (**a**,**d**) or with the coil removed (**b**,**e**). The difference in B_1_^+^ **c** showed a reduction of 2% transmit efficiency with the shim array in place. The difference between the coupling values **f** shows a mean and maximum change of 1% and 5%, respectively

S11 of the loaded RF transmit channels were − 17 dB and − 16 dB before and − 17 dB and − 15 dB after placement of the MC-array. B_1_^+^ mapping in a loading phantom showed an average 2% reduction of B_1_^+^ when the array was attached (see Fig. 2a–c). The noise correlation matrix showed a mean absolute difference in coupling of 1% with a maximum change in coupling of 5% (Fig. 2d–e).

### Simulation-based comparison with existing techniques

**Fig. 3:**
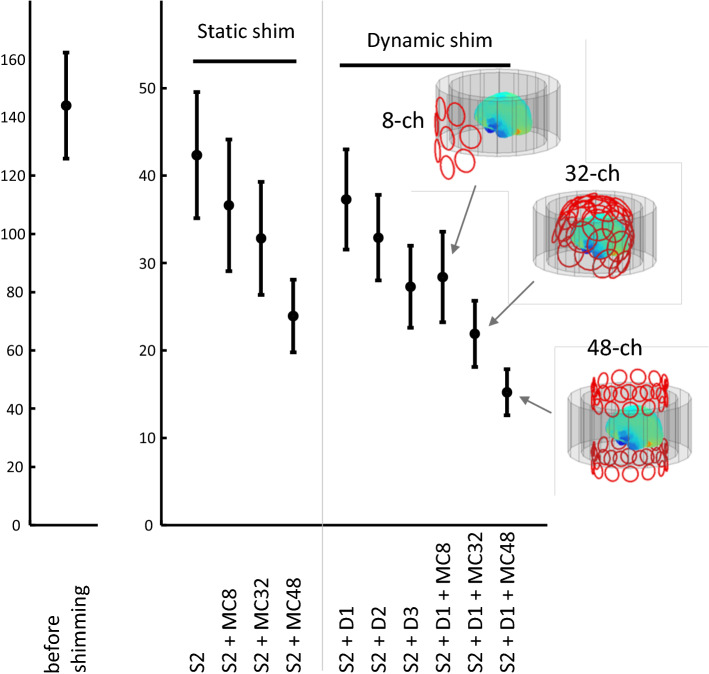
Simulated shimming performance of different approaches over ten subjects. Static second-order shimming (S2) is used as the standard, and all other analysis was performed after removing up to second-order fields from the maps. On top of this, the static eight-channel multi-coil (MC8) array resulted in improve field homogeneity, but did not perform as good as the larger shim arrays with 32- or 48-channels (MC32 and MC48 resp). For dynamic (slice based) shimming, the eight-channel array in combination with dynamic linear shimming (D1) outperformed first and second-order dynamic shimming (D2) and performed not significantly different from dynamic third-order dynamic shimming (D3). Again, dynamic shimming with the larger arrays (MC32 and MC48) performed best

The shimming performance was simulated in ten subjects (Fig. 3). Before shimming, the average field variation in the brain was 144 ± 18 Hz (mean of standard deviations and standard error over subjects). Using the static second-order shimming, whole-brain field variations were reduced to 42 ± 7 Hz. Using the static 8-, 32- and 48-channel MC-arrays the field variation were 37 ± 8 Hz, 35 ± 7 Hz and 33 ± 6 Hz, respectively.

Dynamic first-order SH-shimming, second-order SH-shimming and third-order SH-shimming (in simulation) resulted in whole brain field variations of 37 ± 6 Hz, 33 ± 5 Hz and 27 ± 5 Hz, respectively. Dynamic first-order shimming together with the 8-, 32- and 48- channel MC-arrays resulted in 28 ± 5 Hz, 22 ± 4 Hz and 15 ± 3 Hz, respectively.

Differences between shimming methods were all highly significant (*p* < 0.0003), apart from the comparison between dynamic shimming with 3rd-order spherical harmonics and dynamic shimming with the 8-channel MC-array (*p* = 0.03).

**Fig. 4:**
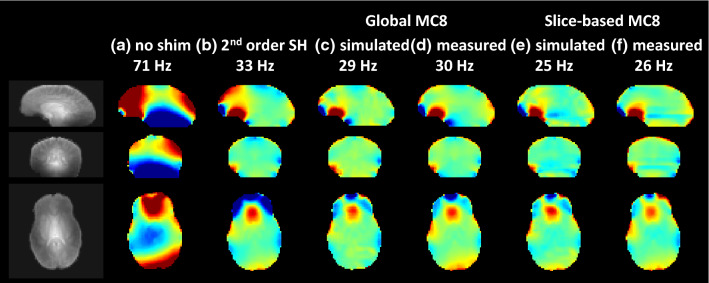
Example of shimming performance on three orthogonal slices (left) in one subject. The field distribution before shim (**a**) of 71 Hz was improved to 33 Hz with second-order spherical harmonic shimming (**b**). Use of the eight-channel MC coil in combination with second-order shimming for whole brain (“static”) shimming improved the shim further to 29 Hz (c, simulated) and 30 Hz (**d**, measured). Slice-based (“dynamic”) MC8 shimming, in combination with static second-order shimming, resulted in 25 Hz (**e**, simulated) and 26 Hz (**f**, measured) standard deviation over the brain

In general, a good similarity was observed between simulated and measured fields (Fig. 4), verifying the applicability of the calibration and shimming algorithm. An example of whole-brain shimming using second-order SH-shimming and the designed eight-channel MC-array is shown in Fig. 4. Note that a per slice optimization on standard deviation in some cases can lead to a local worsening compared to the whole brain shim. This is for example seen as in the middle slices, where the homogeneity in the midbrain is traded off (green to light-blue in Fig. 4b vs c) for improvements in the frontal brain.

### Dynamic shimming for EPI imaging

**Fig. 5:**
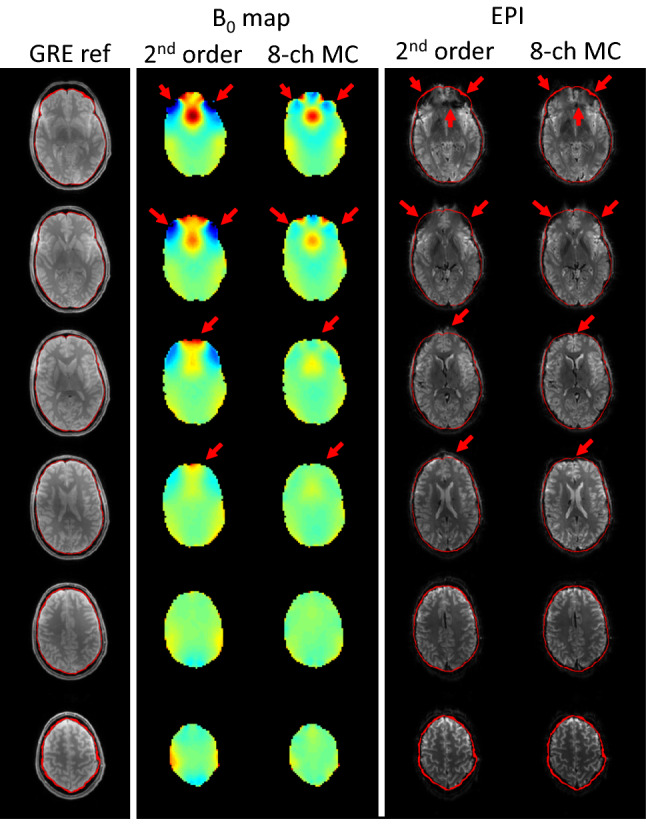
Undistorted 7 T gradient echo (GRE) images as reference (1st column), simulated B0 map with whole brain second-order shimming (2nd column), and B0 map using dynamic shimming with the eight-channel shim array (3rd column, both scaled from − 200 to 200 Hz). Single shot 2 mm multi-slice EPI imaging with standard whole brain second-order shimming (4th column), and single shot EPI imaging using dynamic shimming with the eight-channel shim array (5th column). The EPI image acquired using dynamic shimming shows reduced signal drop-out and reduced image distortions especially in lower brain areas (red arrows)

Dynamic shimming was performed on a fast EPI imaging sequence. Static second-order SH-shimming was compared with the dynamic MC-array and first-order SH-shimming. Figure 5 shows that image distortions and signal dropout is mitigated using the improved B_0_ homogeneity facilitated by dynamic MC-array shimming. Especially in the frontal part of the brain distortions are reduced. For geometric comparison an undistorted gradient echo image is also depicted (red outline). Some minor changes are seen in the more superior slices, indicating some minor local deviations from the predicted field, possibly due to day-to-day variation of the coil position and MR bed positioning accuracy.

## Discussion

MRI and MRS at ultra-high field require additional B_0_ field compensation as compared to lower field strengths. However, all ultra-high field scanners feature a full set of second-order spherical harmonic shims, which is similar to 3 T. For better shimming performance, some systems are equipped with either partial or full third-order shims and, pushing this further, 4th- and 5th-order shimming has been shown. The use of dynamic shimming with higher order shims can provide much higher field homogeneity for multi-slice scans, but this has not been widely implemented, both due to the requirements for a complex eddy current calibration, and lack of control software and hardware as well as possible design constraints to the shim amplifiers. In this work we propose the extension of a traditional shim set with a low-complexity eight-channel shim array that can provide improved field homogeneity compared to standard 2nd order shimming, in both static and dynamic shimming.

The designed eight-channel shim array provides a low-complexity and low-cost approach for improved B_0_ field shimming during ultra-high field brain imaging. Due to head sizes and brain shape differences, differences can be observed between subjects, but the coil provided improved shimming in all cases. In static shimming, used for, e.g., 3D scans, the array improved the whole brain shim by 15% averaged over ten subjects compared to second order shimming. When used for dynamic shimming (used for multi-slice sequences) it improved the whole brain shim by 33% over 10 subjects. This performance is similar to third order dynamic shimming, but without the need for high-order eddy current compensation.

Several groups have previously presented multi-coil shim arrays, where arrays of small loop coils are used instead of whole-body shim coils. Approaches with small amounts of coils have been shown as well as arrays up to 48 coils. However, construction and installation of especially larger multi-coil arrays is technically challenging, and none of the previously presented arrays can be used readily with standard RF hardware at 7 T.

Here, we present a relatively simple multi-coil shim array designed to improve field homogeneity in the human brain using standard 7 T RF coils. The array was designed to balance construction efforts with achievable shim improvements. It facilitates increased B_0_ field homogeneity compared to static spherical harmonic shimming for whole-brain applications, but most importantly enables dynamic shimming for multi-slice sequences for further improvement in B_0_ field homogeneity. The array design is freely available and can be manufactured with minor electronics experience and equipment.

When implementing this setup, care should be taken in the use of the coil. First, care should be taken to position the coil in a reproducible way, as the measured calibration fields are reused for every subject. Here this was done by fixing the array to the transmit coil using brackets, where the RF coil is locked in the bed to ensure similar placement between experiments. In addition, there can be significant forces on the coils during switching, requiring a firm fixture of the coil.

Several choices were made to arrive at a practical design. First, we inferred that the shim coils followed the outside of the RF coil. By this the shim coils were placed outside the RF coil’s built-in shielding, minimizing impact on the RF coil performance. In addition, we optimized the array geometry on a single subject. To validate this choice, we examined a per-subject optimization. This did provide a 1–2 Hz improvement. One subject reached a larger 6 Hz improvement. Overall, we conclude that the single-subject design is applicable to more subjects, especially when considering the complexity of a per-subject design. However, it remains important to be aware of inter-subject variation as caused by varying anatomy and positioning between subjects. Static shimming with the eight-channel MC-array provided improvements in field homogeneity over standard static second-order shimming from 42 ± 7 to 37 ± 8 Hz. On top of that, dynamic shimming with the eight-channel MC-array showed better performance with 28 ± 5 Hz, which was at the level of full dynamic third-order shimming with 27 ± 5 Hz, without the need for complex eddy current compensation generated by dynamic use of higher order SH-coils [25].

As expected, simulations showed that shimming performance scales with MC-array complexity. The close fitting 32-channel MC-array, and particularly the 48-channel MC-array showed improved performance to that of the eight-channel MC-array presented here. However, with geometries, such as the 48-channel design, there is the potential for interference between the shim coils and the RF transmit and/or receive array. This potentially leads to alterations in B_1_-fields, impacting image quality, and E-fields, invalidating SAR and safety considerations assumed of the coil [16]. Although specialized RF coils solutions can alleviate this, this vastly increases the design complexity. Placing the MC-array outside the shield of the RF birdcage coil, as done here, showed negligible loss in RF coil performance [17]. In addition, high element-count arrays require increased complexity in establishing shim amplifiers, cabling, power consumption and heating of the system. In comparison, passive air-cooling was sufficient for the experiments performed in this study. Another technical solution has been shown to be the merge of the RF and shim coil [18, 19]. Using the same conductors for both AC and DC current prevents coupling. However, manufacturing of a suitable RF coil array and interfacing to the system likewise requires significant engineering efforts.

Improved performance may also be obtained by modifying the shape of individual coil elements. Recent work has shown that optimized wire patterns improve performance [26]. Although the construction of an optimized wire pattern is more complex as compared to a multi-loop approach, there is sufficient experience from gradient coil design to allow for robust construction of such coils. This could be a promising solution for high quality shim improvements with a low number of elements.

The impact of the shim array on S-parameters of the NOVA head coil is found to be negligible. This is even further so when considering the putative impact of imprecise coil positioning inherent to the NOVA coil design, where the transmit coil and the anterior part of the receive coil can be slid in the feet-head direction relative to the coil base.

We included the simulated performance of more complex arrays and higher order dynamic shimming as we did not have the hardware available to perform these experiments side-by-side. Care should be taken to compare field homogeneity with other studies, as there are several steps involved in the processing, for example by brain delineation.

We speculate that real-time field updating, to correct for, e.g., breathing induced field fluctuations, could further improve the shim performance in the brain, if the MC-coil fields can provide compensation of the fluctuations of the B_0_ field due to, e.g., breathing.
